# Oral versus intravenous antibiotic treatment of moderate-to-severe community-acquired pneumonia: a propensity score matched study

**DOI:** 10.1038/s41598-024-59026-2

**Published:** 2024-04-09

**Authors:** Anna G. Kaal, Rick Roos, Pieter de Jong, Rianne M. C. Pepping, Johanna M. W. van den Berg, Maarten O. van Aken, Ewout W. Steyerberg, Mattijs E. Numans, Cees van Nieuwkoop

**Affiliations:** 1 Department of Internal Medicine, Haga Teaching Hospital, The Hague, The Netherlands; 2 Department of Biomedical Data Sciences, Leiden University Medical Center, Leiden, The Netherlands; 3 Health Campus The Hague/Department of Public Health and Primary Care, Leiden University Medical Center, The Hague, The Netherlands; 4 Department of Surgery, Albert Schweitzer Hospital, Dordrecht, The Netherlands; 5 Department of Pulmonology, Haga Teaching Hospital, The Hague, The Netherlands

**Keywords:** Outcomes research, Bacterial infection, Respiratory tract diseases

## Abstract

Community-acquired Pneumonia (CAP) guidelines generally recommend to admit patients with moderate-to-severe CAP and start treatment with intravenous antibiotics. This study aims to explore the clinical outcomes of oral antibiotics in patients with moderate-to-severe CAP. We performed a nested cohort study of an observational study including all adult patients presenting to the emergency department of the Haga Teaching Hospital, the Netherlands, between April 2019 and May 2020, who had a blood culture drawn. We conducted propensity score matching with logistic and linear regression analysis to compare patients with moderate-to-severe CAP (Pneumonia Severity Index class III–V) treated with oral antibiotics to patients treated with intravenous antibiotics. Outcomes were 30-day mortality, intensive care unit admission, readmission, length of stay (LOS) and length of antibiotic treatment. Of the original 314 patients, 71 orally treated patients were matched with 102 intravenously treated patients. The mean age was 73 years and 58% were male. We found no significant differences in outcomes between the oral and intravenous group, except for an increased LOS of + 2.6 days (95% confidence interval 1.2–4.0, *p* value < 0.001) in those treated intravenously. We conclude that oral antibiotics might be a safe and effective treatment for moderate-to-severe CAP for selected patients based on the clinical judgement of the attending physician.

## Introduction

Community-acquired pneumonia (CAP) is one of the main causes of mortality and hospitalisation and is usually treated with one or more types of antibiotics^1–3^. CAP has an estimated admission rate of 50–2.940 per 100,000 annually in the adult population of Europe, and is highly dependent of age^4,5^. The costs of CAP are estimated to be around 10.1 billion euros annually in Europe alone, most of which comes from inpatient care^3^.

In current European and American guidelines, treatment location (inpatient or outpatient) and route of antibiotic administration (oral or intravenous) are based on a combination of clinical prediction rules and clinical judgement of the physician^6–10^. These prediction rules (e.g. the Pneumonia Severity Index (PSI) and CURB-65) assess disease severity and divide patients in different categories according to their mortality risk^11,12^. For moderate-to-severe CAP (PSI class III–V, or CURB-65 score ≥ 2), most guidelines advise hospitalisation and treatment with intravenous antibiotics^6–10^.

Some studies have explored the possibility of treating CAP patients with oral antibiotics^13–15^. However, most of these studies did not specify the severity of disease or only included highly selected patient groups with mild-to-moderate CAP. Two studies included hospitalised patients with moderate-to severe CAP and showed similar or even better outcomes for oral treatment compared to intravenous treatment. Both studies did not specify disease severity in patients with treatment failure^14,15^. Furthermore, one study showed that a substantial number of patients presenting to the emergency department (ED) with CAP PSI class IV-V can be treated at home safely^16^.

In this study, we aim to explore the clinical outcomes of oral versus intravenous antibiotic treatment in patients with moderate-to-severe CAP presenting to the ED in an observational cohort of patients representing current daily practice in a large teaching hospital in the Netherlands.

## Materials and methods

### Study design and setting

This is a nested cohort study of a single-centre observational study, the PredictED study. The PredictED study aims to predict bacteraemia in patients presenting to the ED with suspected infection. All patients aged 18 years or older from whom a blood culture was taken during an ED-visit between April 2019 and May 2020 were included. This study was conducted at the ED of the Haga Teaching Hospital in the Netherlands, a large urban hospital with around 600 beds and 50.000 ED-visits annually. The PredictED has been registered at the national trial register: [https://onderzoekmetmensen.nl/nl/trial/23345].

We included patients with an ED diagnosis of moderate-to-severe CAP, defined as PSI class III–V. If patients visited the hospital multiple times, we only included the first visit. We only included patients treated with antibiotics for pneumonia within 24 h of the ED-visit. Furthermore, we excluded patients diagnosed with a COVID-19 infection, an aspiration pneumonia or a hospital-acquired pneumonia (HAP), since these diagnoses require a different treatment approach than for CAP^10^. Aspiration pneumonia was defined as every pneumonia in which, as according to the electronic health record (EHR), the attending physician strongly considered aspiration to be the underlying cause and subsequent empirical treatment was started (according to the local hospital guideline being amoxicillin clavulanic acid or ceftriaxone combined with metronidazole). Furthermore, patients who were incurably ill due to other diseases (e.g. cancer) and opted for palliative care after their ED visit were excluded. Finally, patients who were transferred to other hospitals were excluded.

*Treatment* Treatment of CAP was left to the attending physician at the ED. The physicians followed the local hospital guideline, and could deviate from the first choice in the guideline based on their clinical judgement (e.g. in case of known pathogens from previous positive sputum cultures or suspicion of an atypical respiratory pathogen). Our local hospital guideline recommends treating PSI class III–IV pneumonia with oral amoxicillin 750 mg, 3 times a day (total treatment duration 5 days); or intravenous amoxicillin 1000 mg, 4 times a day (total treatment duration 5 days). The choice for oral or intravenous treatment is based on the clinical judgement of the attending physician at the ED, the local guideline does not contain criteria for clinical judgement. In case of pre-treatment with amoxicillin or failure of amoxicillin treatment, the local guideline recommends an oral switch to doxycycline 100 mg, once a day with a loading dose of 200 mg on the first day (total treatment duration 7 days) or moxifloxacin 400 mg, once a day (total treatment duration 5 days). The first choice of treatment of patients with PSI class V pneumonia is ceftriaxone 2000 mg, once a day (total treatment duration 5 days). In case of clinical suspicion of an atypical respiratory pathogen (e.g. *Legionella* spp.), the addition of a fluoroquinolone is recommended. Based on the pharmacokinetic profiles, our local antibiotic hospital guideline committee considers there to be no rationale for treatment with intravenous amoxicillin or moxifloxacin over oral amoxicillin or moxifloxacin^17–22^. Therefore, our local hospital guideline differs from the Dutch national guideline in the treatment of patients with PSI class III–IV pneumonia as the latter recommends similar antibiotics albeit always intravenously^10^. This difference with the national guideline gave us the unique opportunity to compare oral and intravenous treatment for this patient category.

### Variables and data collection

Demographical, clinical, biochemical, and microbiological data were collected from the EHR. This included the resuscitation code status, describing the type of resuscitation procedures (cardiopulmonary resuscitation, mechanical ventilation, and ICU-admission) used in case of a medical emergency. In case a code status was not documented in the EHR at the time of the ED-visit, two physicians (AK, RR) reviewed the EHRs to evaluate whether there would have been possible reasons to restrict the code status to a non-ICU-admission code.

We defined cerebrovascular disease, congestive heart failure, liver disease, malignancy and renal disease according to the definitions of the Charlson Comorbidity Index^23^. In addition, pulmonary disease was defined as the presence of chronic pulmonary disease including asthma, chronic obstructive pulmonary disease, cystic fibrosis, pulmonary hypertension, or obstructive sleep apnoea. Besides that, immunosuppressant use was defined as the use of medication suppressing the immune system, including the chronic use of glucocorticosteroids, cyclosporine’s, methotrexate, azathioprine, cyclophosphamide, tacrolimus and biologicals. Recent antibiotic use was defined as use of antibiotics up to seven days prior to ED-visit. Finally, we defined altered mental status as a Glasgow Coma Scale < 15 points or a note of altered mental status^24^.

We collected data on antibiotic treatment regimens. Patients were classified as either ‘Oral’ or ‘Intravenous’ according to the method of administration of the first antibiotic regimen. Patients who were given only one dose of intravenous antibiotics and then switched to an oral antibiotic were categorised in the oral treatment group as it is not uncommon at our ED to administer the first dose of antibiotics intravenously before starting oral treatment. Therapeutic antibiotic switches were defined as a change of antibiotic treatment to a more broad-spectrum or entirely different spectrum of antibiotics, or a change from oral to intravenous antibiotics.

Biochemical data consisted of the laboratory test results at the ED required to calculate the PSI-score and inflammatory markers (white blood cell count and C-reactive protein (CRP)). In addition, the presence of pleural effusion on the chest X-ray was documented. Based on these demographical, clinical, and biochemical variables the PSI-score was calculated for all patients.

Microbiological data consisted of all blood and sputum cultures, polymerase chain reactions (PCR) of nasopharyngeal swabs for viral respiratory pathogens, urinary antigen tests and atypical respiratory pathogen tests taken within 48 h of the ED-visit.

### Outcomes

The primary study outcome was 30-day mortality. Secondary study outcomes were ICU-admissions, unplanned readmissions within seven days of discharge, length of hospital stay (LOS) and length of antibiotic treatment.

### Statistical analysis

Descriptive statistics were presented as numbers and percentages, means and standard deviations (SD) or medians and interquartile ranges (IQR), as appropriate. A two-sided *p* value < 0.05 was considered statistically significant.

Missing data was imputed ten times using Multiple Imputation by Chained Equation (MICE)^25^. Rubin’s rules were then used to create a single dataset^26^. Since blood pH is most likely not to be missing at random, we assumed missing values meant a blood pH > 7.35, as it was the clinical judgement of the physician to not perform a blood gas analysis. Since the assignment of oral or intravenous treatment was not random, we performed a propensity score weighted analysis. We estimated the propensity score with a binary logistic regression model consisting of all individual parameters as used in the PSI, using the absolute values where possible, history of pulmonary disease, use of immunosuppressants, recent use of antibiotics, non-ICU code, CRP, peripheral oxygen saturation and the need for oxygen therapy. Matching was done using the nearest neighbour method without replacement, with a 1:2 ratio and a caliper of 0.2 of the SD of the logit of the propensity score^27^. This created a population in which potential confounders were balanced, defined as a standardised mean difference less than 10%^27^. Finally, for the analysis of primary and secondary outcomes we performed Firth’s logistic regression analysis and linear regression analysis on the matched cohort, with correction of the propensity score using a spline function. We performed the LOS analysis for all patients and for only admitted patients.

We conducted multiple post-hoc sensitivity analyses: one among all admitted patients whose diagnosis at discharge remained a respiratory infection and were therefore not misdiagnosed^28^. Other sensitivity analysis consisted of only admitted patients, only non-deceased patients and an analysis using only the patients in the oral group that did not receive a first, intravenous dose of antibiotics at the ED. Finally, we performed regression analysis using traditional correction for confounders, using the same variables as used for the propensity score model.

We reported descriptive outcomes according to multiple subgroups; per PSI class, per bacteremia status, per sepsis status (defined as a qSOFA of 2 or more), per viral coinfection status and according to a level of procalcitonin ≥ 0.25 ng/mL^29^. We reported microbiological outcomes and antibiotic treatments for the matched cohort and the complete cohort*.* Statistical analyses were performed using R Statistical Software version v4.2.2; R Core Team 2022.

### Ethics

The PredictED study was approved by the Medical Ethics Committee and the Institutional Scientific Review Board of the Haga Teaching Hospital (protocol T18‐040). The need for informed consent was waived by The Medical Ethics Committee Leiden The Hague Delft. All methods were performed in accordance with relevant guidelines and regulations. In this study, we adhered to the STROBE reporting guideline for cohort studies.

## Results

**Figure 1:**
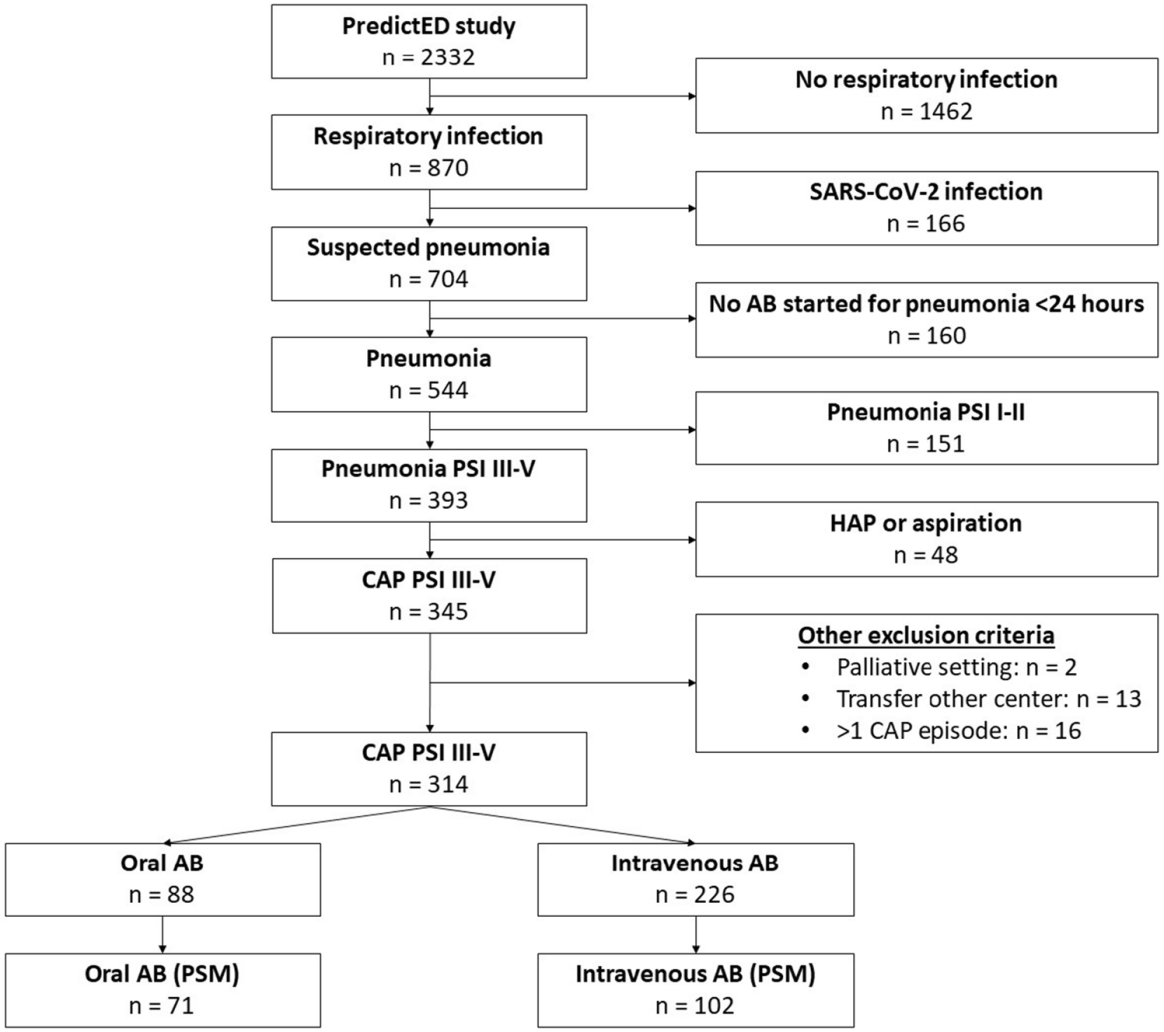
Flowchart of inclusion. *AB* antibiotics, *CAP* community-acquired pneumonia, *HAP* hospital-acquired pneumonia, *PSI* pneumonia severity index, *PSM:* propensity score matching.

Of the 2332 patients presenting to the ED, 314 were diagnosed with a moderate-to-severe CAP. Of those, 88 were treated orally and 226 intravenously (see Fig. 1). After propensity score matching (PSM), 71 patients in the oral group were matched with 102 patients in the intravenous group.

The oral and intravenous group were similar regarding demographics, comorbidities, and code status (Table 1). In the original cohort, more patients in the intravenous group presented with an altered mental status, a higher heart frequency and respiratory rate, and a lower systolic blood pressure and oxygen saturation than the oral group. The median PSI score was higher in the intravenous group (112 vs. 96). 33 out of 76 patients with PSI class III were treated orally (43%) while most patients with PSI class V were treated intravenously (62/67, 93%). The entire intravenous group was hospitalised, while 69% of the patients in the oral group were admitted. After PSM, all variables were balanced across the treatment groups except for systolic blood pressure and recent use of antibiotics (See love plot, Appendix 1).

**Table 1:** Baseline characteristics.

	Unmatched			Matched		
	Oral (n = 88)	Intravenous (n = 226)	aSMD	Oral (n = 71)	Intravenous (n = 102)	aSMD
Demographics						
Age in years, mean (SD)	72 (11)	73 (12)	0.028	73 (11)	73 (12)	0.022
Male, n (%)	50 (56.8)	131 (58.0)	0.023	41 (57.7)	60 (58.8)	0.028
No ICU-admission code, n (%)	31 (35.2)	92 (40.7)	0.112	28 (39.4)	36 (35.3)	0.089
Nursing home resident, n (%)	8 (9.1)	32 (14.2)	0.145	8 (11.3)	14 (13.7)	0.074
Comorbidities						
Cerebrovascular disease, n (%)	15 (17.0)	48 (21.2)	0.090	14 (19.7)	19 (18.6)	0.019
Congestive heart failure, n (%)	17 (19.3)	40 (17.7)	0.042	12 (16.9)	19 (18.6)	0.054
Liver disease, n (%)	2 (2.3)	3 (1.3)	0.083	1 (1.4)	2 (2.0)	0.095
Malignancy, n (%)	25 (28.4)	60 (26.5)	0.042	21 (29.6)	27 (26.5)	0.063
Pulmonary disease, n (%)	39 (44.3)	99 (43.8)	0.010	32 (45.1)	46 (45.1)	0.028
Renal disease, n (%)	9 (10.2)	31 (13.7)	0.101	9 (12.7)	12 (11.8)	0.093
CCI, median [IQR]	4 [3–6]	5 [3–6]	0.043	5 [3–6]	4 [3–6]	0.060
Immunosuppressant use, n (%)	17 (19.3)	38 (16.8)	0.067	14 (19.7)	19 (18.6)	0.018
Recent antibiotic use, n (%)	29 (33.0)	60 (26.5)	0.145	25 (35.2)	26 (25.5)	0.150
Physical examination						
Altered mental status, n (%)	3 (3.4)	47 (20.8)	0.428	3 (4.2)	5 (4.9)	0.039
HF (beats/minute), mean (SD)	102 (18)	110 (24)	0.306	102 (19)	106 (22)	0.090
Oxygen saturation (%), median [IQR]	94 [91–96]	93 [90–95]	0.293	94 [91–96]	94 [92–96]	0.038
Oxygen therapy, n (%)	11 (12.5)	75 (33.2)	0.439	11 (15.5)	18 (17.6)	0.064
RR (breaths/minute), median [IQR]	22 [18–26]	26 [20–30]	0.368	22 [18–26]	24 [18–30]	0.032
SBP (mmHg), mean (SD)	138 (29)	129 (30)	0.317	141 (29)	137 (28)	0.166
Temperature (°C), mean (SD)	38.4 (0.7)	38.6 (1.1)	0.159	38.5 (0.6)	38.6 (1.0)	0.081
Additional diagnostics						
Blood pH, median [IQR]	7.46 [7.45–7.47]	7.46 [7.43,7.48]	0.184	7.46 [7.44–7.46]	7.46 [7.44–7.47]	0.052
CRP (mg/L), median [IQR]	67 [34–170]	125 [47–268]	0.370	76 [38–178]	109 [44–196]	0.030
Glucose (mmol/L), median [IQR]	7.1 [6.2–8.7]	7.6 [6.6–9.4]	0.233	7.3 [6.4–8.9]	7.3 [6.2–9.0]	0.011
Hematocrit < 30%, n (%)	6 (6.8)	20 (8.8)	0.072	4 (5.6)	7 (6.9)	0.000
Sodium (mmol/L), median [IQR]	137 [135–138]	135 [132–138]	0.316	136 [134–138]	136 [133–138]	0.068
Urea (mmol/L), median [IQR]	6.1 [4.9–7.7]	7.8 [5.8–11.7]	0.447	6.6 [5.1–8.7]	6.2 [4.9–8.8]	0.036
WBCs (n × 10^9/L), median [IQR]	11.8 [8.7–15.2]	12.8 [8.8–17.8]	0.280	11.9 [9.4–15.4]	12.7 [8.9–16.6]	0.183
pO2 < 60 mmHg or SaO2 < 90%, n (%)	20 (22.7)	58 (25.7)	0.067	14 (19.7)	21 (20.6)	0.034
Pleural effusion on X-ray, n (%)	16 (18.2)	48 (21.2)	0.075	13 (18.3)	19 (18.6)	0.000
Pneumonia severity index (PSI)						
PSI class						
III, n (%)	33 (37.5)	43 (19.0)		22 (31.0)	26 (25.5)	
IV, n (%)	50 (56.8)	121 (53.5)		44 (62.0)	62 (60.8)	
V, n (%)	5 (5.7)	62 (27.4)		5 (7.0)	14 (13.7)	
PSI score, median [IQR]	96 [85–109]	112 [94–133]	0.748	97 [87–110]	103 [90–118]	0.219
Outcomes						
30-day mortality, n(%)	4 (4.5)	33 (14.6)		4 (5.6)	8 (7.8)	
Admission, n (%)	61 (69.3)	226 (100.0)		49 (69.0)	102 (100.0)	
ICU-admission, n (%)	1 (1.1)	28 (12.4)		1 (1.4)	6 (5.9)	
Readmission, n (%)	5 (5.7)	17 (7.5)		4 (5.6)	9 (8.8)	

Baseline characteristics before and after propensity score matching. Variables with an aSMD lower than 0.10 are considered to be balanced. *aSMD* absolute standardised mean difference, *CCI* charlson comorbidity index, *CRP* C-reactive protein, *HF* heart frequency, *ICU* intensive care unit, *IQR* interquartile range, *RR* respiratory rate, *SBP* systolic blood pressure, *SD* standard deviation, *WBC* white blood cells. The number of missing values for continuous variables in the unmatched oral group were RR (n = 6), SBP (n = 2), glucose (n = 1) and blood pH (n = 31), and for the unmatched intravenous group these were: RR (n = 7), glucose (n = 3), sodium (n = 1), urea (n = 3) and blood pH (n = 57).

### Primary outcome

In total, 37/314 patients (11.8%) died during this study, 33 of whom were treated intravenously (Table 1, Appendix 2). In the propensity score matched population, 4/71 patients (5.6%) died in the oral group and 8/102 patients (7.8%) in the intravenous group. Three out of four patients in the oral group died due to causes related to the pneumonia, while the other patient died due to progression of lung cancer. All eight patients in the intravenous group died due to causes related to the pneumonia. We did not find a difference in mortality for intravenous versus oral treatment (aOR 1.1 (0.3–4.7), *p* = 0.84, Table 2).

**Table 2:** Clinical outcomes of oral versus intravenous treatment for patients with CAP.

A	Oral (n = 71)	Intravenous (n = 102)	OR	95% CI	*p* value	aOR	95% CI	*p* value
Mortality, n (%)	4 (5.6)	8 (7.8)	1.2	0.4–5.0	0.76	1.1	0.3–4.7	0.84
ICU-admission, n (%)	1 (1.4)	6 (5.9)	3.0	0.6–80.1	0.23	2.8	0.6–73.2	0.23
Readmission within 7 days, n (%)	4 (4.6)	9 (8.8)	1.7	0.6–6.9	0.36	1.7	0.5–6.8	0.37

B	Oral (n = 71)	Intravenous (n = 102)	β	95% CI	*p* value	aβ	95% CI	*p* value
Length of stay (days), median [IQR]^a^	2 [0–4]	4 [2–6]	2.6	1.2–4.0	< 0.001	2.6	1.2–4.0	< 0.001
Length of stay (days), median [IQR]^b^	3 [2–6]	4 [2–6]	1.3	− 0.3 to 3.0	0.12	1.4	− 0.3 to 3.0	0.10
Length of antibiotic treatment (days), median [IQR]	7 [5–7.5]	6.5 [5.3–9]	2.1	− 0.2 to 4.4	0.08	2.2	− 0.1 to 4.6	0.06

(A) Logistic regression (B) Linear regression. *CI* confidence interval, *ICU* intensive care unit, *OR* odds ratio, *β = effect size in days. aOR and Aβ* odds ratio and effect size adjusted for the propensity score. aOR Adjusted odds ratio, *CI* confidence interval, *ICU* intensive care unit, *OR* odds ratio. ^a^Outcomes for only non-deceased patients: 67 treated orally, 93 treated intravenously. ^b^Outcomes for only hospitalised, non-deceased patients: 45 patients treated orally, 93 treated intravenously.

### Secondary outcomes

A total of 29/314 patients (9.2%) were admitted to the ICU and 22 patients (7.0%) were readmitted to the hospital within seven days (Table 1, Appendix 2). No significant differences were seen in ICU-admission rate (aOR 2.8 (0.6–73.2), *p* = 0.23) and readmission rate (aOR 1.7 (0.5–6.8), *p* = 0.37) between both PSM groups (Table 2).

In the matched study population, LOS was significantly longer (4 days vs. 2 days) in the intravenous group (adjusted coefficient: 2.6 days (1.2–4.0), *p* < 0.001). When limiting the analysis to hospitalised patients, no significant difference in LOS was found (adjusted coefficient: 1.4 days (− 0.3 to 3.0), *p* = 0.10). Finally, length of antibiotic treatment did not differ significantly between the oral and intravenous group (7.0 days vs. 6.5 days, adjusted coefficient: 2.2 days (− 0.1 to 4.6), *p* = 0.06).

Mortality increased with every PSI class for both groups (Table 3, Appendix 2). In the matched intravenous group, 6/88 (6.8%) patients with a PSI class III/IV were admitted to the ICU compared to 1/66 (1.5%) in the matched oral group. No orally treated patients with a PSI V were readmitted to our hospital.

**Table 3:** Primary outcomes per PSI-class.

	Oral (n = 71)	Intravenous (n = 102)
Mortality, n (%)		
PSI III	0/22 (0.0)	0/26 (0.0)
PSI IV	3/44 (6.8)	6/62 (9.7)
PSI V	1/5 (20.0)	2/14 (14.3)
ICU-admission, n (%)		
PSI III	0/22 (0.0)	1/26 (3.8)
PSI IV	1/44 (2.3)	5/62 (8.1)
PSI V	0/5 (0.0)	0/14 (0.0)
Readmission within 7 days, n (%)		
PSI III	1/22 (4.5)	2/26 (7.7)
PSI IV	3/44 (6.8)	7/62 (11.3)
PSI V	0/5 (0.0)	0/14 (0.0)
Length of stay (days), median [IQR]		
PSI III	1 [0–2.8]	3 [2–4.8]
PSI IV	2 [0–5]	4 [3–7]
PSI V	2.5 [0.8–5]	4.5 [2.8–7]
Length of stay (days)^a^, median [IQR]		
PSI III	2 [1–4]	3 [2–4.8]
PSI IV	3 [2–6]	4 [3–7]
PSI V	4 [2.5–6]	4.5 [2.8–7]
Length of antibiotic treatment (days), median [IQR]		
PSI III	7 [5–7]	6.5 [6–7.8]
PSI IV	7 [5–8]	7 [5–9]
PSI V	7 [5–7]	6 [5.3–8]

*ICU* intensive care unit, *IQR* interquartile range, *PSI* pneumonia severity index. ^a^Only hospitalised patients.

All post-hoc sensitivity analyses showed similar results for all primary and secondary outcomes (Appendices 3–7). Descriptive outcomes according to the predefined subgroups can be found in Appendices 8–11.

### Microbiology

In the matched study population, blood cultures were positive in 3/71 patients in the oral group (4.2%) and 12/102 patients in the intravenous group (11.8%). In blood cultures, the most common respiratory pathogen was *Streptococcus pneumoniae*. Sputum cultures were performed in 29/71 orally treated patients (41%) and 44/102 intravenously treated patients (43%). In the sputum cultures, the most common respiratory pathogen in both groups was *Haemophilus influenzae*. Viral swabs were performed in 40/71 orally treated patients and 54/102 intravenously treated patients, with *Influenza A* being the most common pathogen in the oral group (n = 3), while *Rhinovirus* (n = 4) was most common pathogen in the intravenous group. Urine antigen tests were more often performed in intravenously treated patients (34% vs. 14%) (Appendices 12–19).

### Antibiotic treatment

In the matched study population, amoxicillin (31/71), doxycycline (19/71), moxifloxacin (13/71) and amoxicillin clavulanic acid (6/71) were the most common antibiotics in the oral group; while amoxicillin (38/102), ceftriaxone (27/102), cefuroxime (15/102) and amoxicillin clavulanic acid (11/102) were most common in the intravenous group (Appendix 21). In the oral group, 5 out of 71 patients (7%) switched from oral to intravenous antibiotics (median switch time 1 day^1^). In the oral group, 9 patients received a single intravenous dose at the ED (2 received amoxicillin, 6 ceftriaxone, and 1 ceftriaxone and tobramycin) before starting oral therapy. In the intravenous group, 84/102 patients (82%) switched to oral antibiotics (median switch time 2 days^1–3^). Of these patients, 72/84 (86%) switched within three days (Appendix 23).

## Discussion

In this study, we explored the safety and efficacy of oral versus intravenous antibiotics as prescribed in current daily practice in patients with moderate-to-severe CAP (PSI class III–V) at the ED in a large teaching hospital in the Netherlands. We found no significant differences in 30-day mortality rate, ICU-admissions or readmissions. We observed that intravenous treatment was associated with an extra 2.6 days of admission (95% CI (1.2–4.0), *p* < 0.001) compared with oral treatment. This suggests that oral treatment, when based on clinical judgement of the ED-physician, might be a reasonable treatment option for patients with moderate-to-severe CAP.

The mortality rate for the complete (unmatched) cohort was higher than expected based on the PSI score, specifically for those treated intravenously. This is most likely the result of only including patients for whom a blood culture was performed. Conversely, orally treated patients showed a lower or similar mortality rate than expected. This difference may mainly reflect the clinical judgement of the treating physician. For instance, the physician decided to treat the patient intravenously even when the PSI suggested moderate CAP. For the PSI class V patients who were treated orally, the assessment was made that oral treatment would be sufficient despite classification as severe CAP. This is also reflected in the differences in vital functions at baseline. Gut feeling, an important factor in clinical judgement, was found to be the second most important factor in identifying sepsis in a study that aimed to gain insight in the clinical decision making in a population of general practitioners^30^. This supports our hypothesis that clinical judgement is an important factor in assessing severity of illness and choosing the appropriate method of administration.

In the oral group, 31% were treated as outpatients. In times when healthcare systems have increasingly limited capacity for inpatient care, exploring treatment options that can be given outside the hospital are of great importance. Interestingly, we found no difference in LOS between oral and intravenous treatment for those admitted to the hospital. This might partly be explained by the fact that 82% of our intravenously treated patients were switched to oral treatment, of which 86% switched within three days, which is considered to be an early switch in literature^31^. Such an early switch is associated with a reduction in length of hospital stay of two days in patients with severe CAP^31^.

In this study, 10 out of the 88 patients treated orally received one dose of intravenous antibiotics before starting oral treatment. As it is known that such a dose has a big impact on the bacterial load of sensitive micro-organisms, one might argue that our oral group is actually not a strictly oral group^32–34^. We did allow for this approach, since one intravenous dose would still allow further treatment at home instead of hospital admission. The sub-analysis comparing only patients with strictly oral treatment versus intravenous treatment did not lead to different conclusions.

Oral treatment of CAP has already been widely evaluated and implemented for the treatment of mild-to-moderate cases^6–10,13^. However, the role of oral antibiotics in the upfront treatment of more severe cases has not been sufficiently assessed despite the high bioavailability of oral antibiotics like amoxicillin, doxycycline and moxifloxacin^17–19,35^. Only two studies with a large majority of moderate-to-severe CAP patients showed similar or even better outcomes for oral treatment compared to intravenous treatment, but did not specify the disease severity for patients with treatment failure^14,15^. Furthermore, a large observational study in Canada showed that 13.7% of CAP PSI class IV and V patients could safely be managed as outpatients^16^. Meanwhile, a recent large study showed that the vast majority of CAP patients are still treated with intravenous antibiotics, and early switch only happened in 6% of the patients^36^. Our study adds to the evidence that the role of oral antibiotics could likely be increased in the management of moderate-to-severe-CAP.

A strength of this study is that it is an accurate representation of daily ED practice in a heterogeneous, real-life population. Furthermore, we believe that the quality of the data collection was excellent, with almost no missing data. However, our study also has limitations. First, this study describes a relatively small population limited to one hospital, which might lead to concerns regarding the power to detect relevant differences. Therefore, we emphasise that this is an exploratory study, and our results should not be seen as prove for non-inferiority of oral versus intravenous treatment. Furthermore, antibiotic treatment regimens will differ according to local epidemiology and resistance patterns, so extrapolating these results to other regions should be done with caution. Third, this study is susceptible to information bias, as we were only able to use the available information in the EHR. Hypothetically, although it would be unlikely to differ between the two groups, patients could have been readmitted to other hospitals without our knowledge. Although we applied state-of-the-art statistical methods such as propensity score matching and multiple imputation to balance differences between the orally and intravenously treated patients, there is a risk of residual confounding. We hypothesise that this residual confounding is mainly caused by clinical judgement, which would be acceptable since this study was designed to explore the outcomes of patient who were given oral treatment based on clinical judgement. However, there is a possibility that other factors have led to residual confounding that we have not taken into account. Finally, another limitation is the fact that we do not have a clear definition for clinical judgement, due to the retrospective nature of the study. We believe that clinical judgment or gut feeling plays a big role in identifying patients suitable for oral treatment, and future studies should carefully consider how to take clinical judgement into account.

## Conclusion

Oral antibiotics appear to be a safe and effective treatment of moderate-to-severe CAP for selected patients based on clinical judgement of the attending physician. The dogma ‘oral is the new iv’^37^ should be the topic of future studies on moderate-to-severe CAP patients, with the aim to avoid overtreatment, and thereby improving antibiotic stewardship, and unnecessary hospitalisations.

### Supplementary Information

Supplementary Information.

## Data Availability

The datasets used in this study are not publicly available as they contain health related data. Limited datasets without any identifiable data are available from the corresponding author upon reasonable request.
