# Supplementary material for: Oral versus intravenous antibiotic treatment of moderate-to-severe community-acquired pneumonia: a propensity score matched study
Source: Sci Rep. 2024 Apr 9;14:8271. doi: 10.1038/s41598-024-59026-2 (PMC11004140; doi:10.1038/s41598-024-59026-2)
Supplement: Supplementary file 1 — Supplementary Information. [file 41598_2024_59026_MOESM1_ESM.docx]

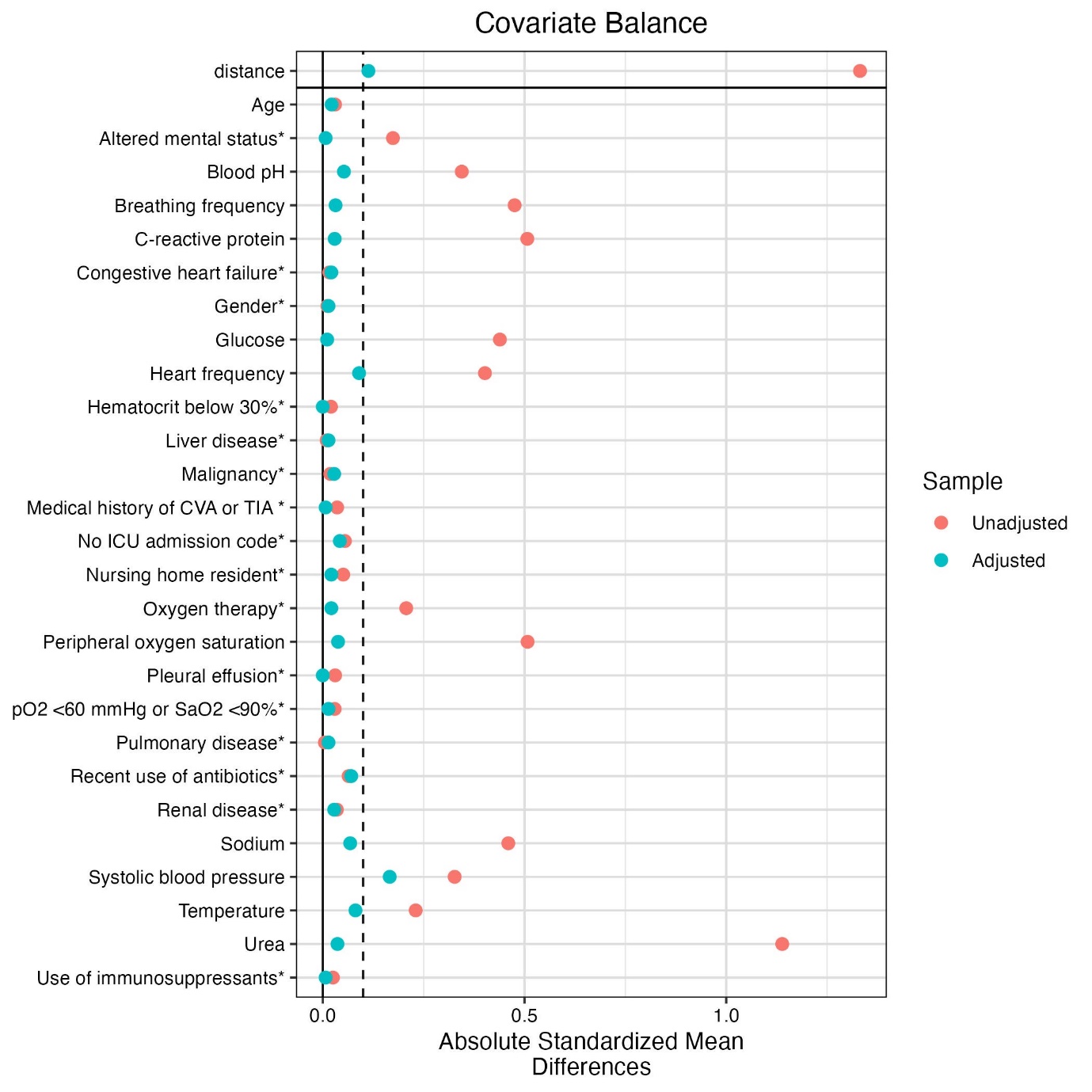


*Appendix 1:* Love plot showing the absolute standardised mean differences before (Unadjusted) and after (adjusted) matching for all variables used to calculate the propensity score. Variables with an asterisk (*) represent binary variables. Variables below the dotted line (<0.1) are considered to be balanced.

*Appendix 2:* Primary outcomes per PSI-class for the complete cohort. *:* ICU: Intensive care unit; IQR: Interquartile range; PSI: Pneumonia severity index. ^a^ Only hospitalised patients.

|  | Oral (n = 88) | Intravenous (n = 226) |
| --- | --- | --- |
| Mortality, n (%) |  |  |
| PSI III | 0/33 (0.0) | 3/43 (7.0) |
| PSI IV | 3/50 (6.0) | 16/121 (13.2) |
| PSI V | 1/5 (20.0) | 14/62 (22.6) |
| ICU-admission, n (%) |  |  |
| PSI III | 0/33 (0.0) | 6/43 (14.0) |
| PSI IV | 1/50 (2.0) | 12/121 (9.9) |
| PSI V | 0/5 (0.0) | 10/62 (16.1) |
| Readmission within 7 days, n (%) |  |  |
| PSI III | 1/33 (3.3) | 2/43 (4.7) |
| PSI IV | 4/50 (8.0) | 11/121 (9.1) |
| PSI V | 0/5 (0.0) | 4/62 (6.5) |
| Length of stay (days), median [IQR] |  |  |
| PSI III | 1 [0.0-3.0] | 3.5 [2.0-5.0] |
| PSI IV | 2 [0.0-5.0] | 5 [3.0-7.0] |
| PSI V | 2.5 [0.8-5.0] | 6.5 [3.0-11.5] |
| Length of stay (days)^a^, median [IQR] |  |  |
| PSI III | 2.5 [1.0-4.8] | 3.5 [2.0-5.0] |
| PSI IV | 3.5 [2.0-6.0] | 5.0 [3.0-7.0] |
| PSI V | 4.0 [2.5-6.0] | 6.5 [3.0-11.5] |
| Length of antibiotic treatment (days), median [IQR] |  |  |
| PSI III | 7.0 [5.0-7.0] | 7.0 [6.0-8.5] |
| PSI IV | 7.0 [5.0-7.0] | 7.0 [5.0-9.0] |
| PSI V | 7.0 [5.0-7.0] | 6.0 [5.0-10.0] |

*Appendix 3*: Sensitivity analysis of clinical outcomes of oral versus intravenous treatment for patients with CAP by only including all admitted patients, analysed with propensity score matching. **A** Logistic Firth’s regression **B** Linear regression. Adjusted for propensity score. aOR: Adjusted odds ratio; CI: Confidence interval; ICU: Intensive care unit; OR: Odds ratio. ^a^Outcomes for only hospitalised, non-deceased patients: 56 patients treated orally, 81 treated intravenously.

| A | Oral  (n = 60) | Intravenous (n = 91) | OR | 95% CI | p-value | aOR | 95% CI | p-value |
| --- | --- | --- | --- | --- | --- | --- | --- | --- |
| Mortality, n (%) | 4 (6.7) | 9 (9.9) | 1.3 | 0.4-5.5 | 0.64 | 1.2 | 0.4-5.1 | 0.75 |
| ICU-admission, n (%) | 1 (1.7) | 4 (4.4) | 2.3 | 0.4-60.8 | 0.38 | 2.1 | 0.4-55.6 | 0.39 |
| Readmission within 7 days, n (%) | 2 (3.3) | 9 (9.9) | 3.0 | 0.8-23.5 | 0.14 | 2.9 | 0.8-24.2 | 0.13 |

| B | Oral  (n = 60) | Intravenous (n = 91) | β | 95% CI | p-value | aβ | 95% CI | p-value |
| --- | --- | --- | --- | --- | --- | --- | --- | --- |
| Length of stay (days), median [IQR]^a^ | 3 [1-6] | 4 [2-6] | 1.1 | -0.4-2.6 | 0.16 | 1.1 | 0.5-2.7 | 0.17 |
| Length of antibiotic treatment (days), median [IQR] | 7 [5-8] | 7 [5-8] | 1.7 | -0.9-4.3 | 0.19 | 1.8 | -0.8-4.4 | 0.18 |

*Appendix 4*: Sensitivity analysis of clinical outcomes of oral versus intravenous treatment for patients with CAP by only including those whose diagnosis at discharge remained a respiratory infection and were therefore not misdiagnosed, analysed with propensity score matching. **A** Logistic Firth’s regression **B** Linear regression. Adjusted for propensity score. aOR: Adjusted odds ratio; CI: Confidence interval; ICU: Intensive care unit; OR: Odds ratio. ^a^ Outcomes for only hospitalised, non-deceased patients: 49 patients treated orally, 71 treated intravenously.

| A | Oral  (n = 53) | Intravenous (n = 81) | OR | 95% CI | p-value | aOR | 95% CI | p-value |
| --- | --- | --- | --- | --- | --- | --- | --- | --- |
| Mortality, n (%) | 4 | 9 | 1.2 | 0.4-5.0 | 0.76 | 1.2 | 0.4-5.0 | 0.80 |
| ICU-admission, n (%) | 1 | 6 | 2.7 | 0.5-71.1 | 0.29 | 2.6 | 0.5-79.4 | 0.28 |
| Readmission within 7 days, n (%) | 2 | 5 | 1.8 | 0.5-14.6 | 0.44 | 1.7 | 0.4-14.3 | 0.45 |

| B | Oral  (n = 53) | Intravenous (n = 81) | β | 95% CI | p-value | aβ | 95% CI | p-value |
| --- | --- | --- | --- | --- | --- | --- | --- | --- |
| Length of stay (days), median [IQR]^a^ | 3 [1-6] | 4 [3-7] | 1.6 | 0-3.3 | 0.05 | 1.6 | -0.1-3.3 | 0.06 |
| Length of antibiotic treatment (days), median [IQR] | 7 [5-8] | 6 [5-9] | 2.0 | -0.8-4.8 | 0.16 | 2.1 | -0.8-4.9 | 0.15 |

*Appendix 5:* Sensitivity analysis for ICU-admission, readmission and length of antibiotic treatment for oral versus intravenous treatment for patients with CAP by excluding all deceased patients within 30 days of presentation. **A** Logistic Firth’s regression **B** Linear regression. Adjusted for the propensity score. aOR: adjusted odds ratio; CI: Confidence interval; ICU: Intensive care unit; OR: Odds ratio.

| A | Oral  (n = 64) | Intravenous (n = 93) | OR | 95% CI | p-value | aOR | 95% CI | p-value |
| --- | --- | --- | --- | --- | --- | --- | --- | --- |
| ICU-admission, n (%) | 0 (0.0) | 4 (4.3) | 5.9 | 0.6-Inf | 0.24 | 5.4 | 0.6-Inf | 0.20 |
| Readmission within 7 days, n (%) | 4 (6.3) | 9 (9.7) | 1.6 | 0.5-6.5 | 0.44 | 1.5 | 0.5-6.3 | 0.47 |

| B | Oral  (n = 64) | Intravenous (n = 93) | β | 95% CI | p-value | aβ | 95% CI | p-value |
| --- | --- | --- | --- | --- | --- | --- | --- | --- |
| Length of antibiotic treatment (days), median [IQR] | 7 [5-7.3] | 7 [6-9] | 2.5 | -0.1-5.0 | 0.05 | 2.5 | -0.1-5.0 | 0.06 |

*Appendix 6***:** Sensitivity analysis of clinical outcomes of oral versus intravenous treatment for patients with CAP by excluding all patients with only one intravenous administration of antibiotics analysed with propensity score matching. **A** Logistic Firth’s regression **B** Linear regression. Adjusted for the propensity score. aOR: Adjusted odds ratio; CI: Confidence interval; ICU: Intensive care unit; OR: Odds ratio. ^a^ Outcomes for only non-deceased patients: 59 treated orally, 78 treated intravenously. ^b^Outcomes for only hospitalised, non-deceased patients: 41 patients treated orally, 78 treated intravenously.

| A | Oral  (n = 61) | Intravenous (n = 89) | OR | 95% CI | p-value | aOR | 95% CI | p-value |
| --- | --- | --- | --- | --- | --- | --- | --- | --- |
| Mortality, n (%) | 2 (3.3) | 10 (11.2) | 2.5 | 0.7-19.8 | 0.22 | 2.4 | 0.6-19.1 | 0.23 |
| ICU-admission, n (%) | 1 (1.6) | 9 (10.1) | 3.8 | 0.8-101.3 | 0.14 | 3.8 | 0.8-104.7 | 0.12 |
| Readmission within 7 days, n (%) | 4 (6.6) | 8 (9.0) | 1.5 | 0.5-6.0 | 0.53 | 1.4 | 0.5-5.9 | 0.55 |

| B | Oral  (n = 61) | Intravenous (n = 89) | β | 95% CI | p-value | aβ | 95% CI | p-value |
| --- | --- | --- | --- | --- | --- | --- | --- | --- |
| Length of stay (days), median [IQR]^a^ | 2 [0-4] | 4 [2-6.8] | 2.9 | 1.2-4.5 | <0.001 | 2.8 | 1.2-4.4 | <0.001 |
| Length of stay (days), median [IQR]^b^ | 3 [1-5] | 4 [2-6.8] | 1.6 | -0.3-3.5 | 0.09 | 1.8 | -0.1-3.7 | 0.06 |
| Length of antibiotic treatment (days), median [IQR] | 7 [5-7] | 5 [5-10] | 2.4 | -0.3-5.0 | 0.09 | 2.4 | -0.3-5.1 | 0.08 |

*Appendix 7*: Clinical outcomes of oral versus intravenous treatment for patients with CAP using traditional regression analysis. **A** Logistic regression **B** Linear regression. Adjusted for all individual parameters of the pneumonia severity index, history of pulmonary disease, recent antibiotic use, immunosuppressant use, no intensive care unit admission code, C-reactive protein, peripheral oxygen saturation and use of oxygen therapy. aOR: Adjusted odds ratio; CI: Confidence interval; ICU: Intensive care unit; OR: Odds ratio. ^a^ Outcomes for only non-deceased patients: 84 treated orally, 191 treated intravenously. ^b^ Outcomes for only hospitalised, non-deceased patients: 57 patients treated orally, 191 treated intravenously.

| A | Oral  (n = 88) | Intravenous (n = 226) | OR | 95% CI | p-value | aOR | 95% CI | p-value |
| --- | --- | --- | --- | --- | --- | --- | --- | --- |
| Mortality, n (%) | 4 (4.5) | 33 (14.6) | 3.6 | 1.2-10.5 | 0.01 | 1.7 | 0.6-8.4 | 0.36 |
| ICU-admission, n (%) | 1 (1.1) | 28 (12.4) | 12.3 | 1.6-91.9 | 0.002 | 3.0 | 0.6-114.8 | 0.18 |
| Readmission within 7 days, n (%) | 5 (5.7) | 17 (7.5) | 1.4 | 0.5-3.8 | 0.57 | 1.4 | 0.5-5.8 | 0.49 |

| B | Oral  (n = 88) | Intravenous (n = 226) | β | 95% CI | p-value | aβ | 95% CI | p-value |
| --- | --- | --- | --- | --- | --- | --- | --- | --- |
| Length of stay (days), median [IQR] | 1.5 [0.0-4.0] | 5.0 [3.0-7.0] | 3.6 | 2.1-5.1 | <0.001 | 1.8 | 0.3-3.3 | 0.02 |
| Length of stay (days), median [IQR]^a^ | 3.0 [1.0-6.0] | 5.0 [3.0-7.0] | 2.2 | 0.5-4.0 | 0.01 | 0.8 | -0.9-2.4 | 0.37 |
| Length of antibiotic treatment (days), median [IQR] | 7.0 [5.0-7.0] | 6.5 [5.0-9.0] | 1.0 | -0.6-2.6 | 0.23 | 1.1 | -0.7-2.8 | 0.23 |

*Appendix 8***:** Primary outcomes stratified per bacteremia status. AB: Antibiotic; ICU: Intensive care unit; IQR: Interquartile range; PSI: Pneumonia severity index. ^a^ Only hospitalised patients.

|  | Oral (n = 88) | Intravenous (n = 226) |
| --- | --- | --- |
| Mortality, n (%) |  |  |
| Bacteremia | 0/3 (0.0) | 4/26 (15.4) |
| No bacteremia | 4/85 (4.7) | 29/200 (14.5) |
| ICU-admission, n (%) |  |  |
| Bacteremia | 0/3 (0.0) | 8/26 (30.8) |
| No bacteremia | 1/85 (1.2) | 20/200 (10.0) |
| Readmission within 7 days, n (%) |  |  |
| Bacteremia | 0/3 (0.0) | 4/26 (15.4) |
| No bacteremia | 5/85 (5.9) | 13/200 (6.5) |
| Length of stay (days), median [IQR] |  |  |
| Bacteremia | 12 [6.5-13.5] | 6.5 [4.0-8.8] |
| No bacteremia | 1 [0.0-4.0] | 4.0 [3.0-7.0] |
| Length of stay (days)^a^, median [IQR] |  |  |
| Bacteremia | 12 [6.5-13.5] | 6.5 [4.0-8.8] |
| No bacteremia | 3.0 [1.3-5.8] | 4.0 [3.0-7.0] |
| Length of antibiotic treatment (days), median [IQR] |  |  |
| Bacteremia | 15.0 [13.5-15.5] | 7.5 [6.3-12.3] |
| No bacteremia | 7.0 [5.0-7.0] | 6.0 [5.0-9.0] |

*Appendix 9:* Primary outcomes stratified per proven viral coinfection. AB: Antibiotic; ICU: Intensive care unit; IQR: Interquartile range; PSI: Pneumonia severity index. ^a^ Only hospitalised patients.

|  | Oral (n = 88) | Intravenous (n = 226) |
| --- | --- | --- |
| Mortality, n (%) |  |  |
| Viral infection | 1/11 (9.1) | 3/35 (8.6) |
| No viral infection | 3/77 (3.9) | 30/191 (15.7) |
| ICU-admission, n (%) |  |  |
| Viral infection | 0/11 (0.0) | 10/35 (28.6) |
| No viral infection | 1/77 (1.3) | 18/191 (9.4) |
| Readmission within 7 days, n (%) |  |  |
| Viral infection | 0/11 (0.0) | 2/35 (5.7) |
| No viral infection | 5/77 (6.5) | 15/191 (7.9) |
| Length of stay (days), median [IQR] |  |  |
| Viral infection | 1.5 [1-7] | 4.5 [3-10.3] |
| No viral infection | 1.5 [0-4] | 5 [3-7] |
| Length of stay (days)^a^, median [IQR] |  |  |
| Viral infection | 2 [1-7] | 4.5 [3-10.3] |
| No viral infection | 3 [2-5.3] | 5 [3-7] |
| Length of antibiotic treatment (days), median [IQR] |  |  |
| Viral infection | 7 [5-8.5] | 6 [5-9] |
| No viral infection | 7 [5-7] | 7 [5-9] |

*Appendix 10:* Primary outcomes stratified per sepsis status defined as a qSOFA score of 2 or higher. AB: Antibiotic; ICU: Intensive care unit; IQR: Interquartile range; PSI: Pneumonia severity index. ^a^ Only hospitalised patients.

|  | Oral (n = 88) | Intravenous (n = 226) |
| --- | --- | --- |
| Mortality, n (%) |  |  |
| Septic | 1/6 (16.7) | 10/52 (19.2) |
| Non-septic | 3/82 (3.7) | 23/174 (13.2) |
| ICU-admission, n (%) |  |  |
| Septic | 0/6 (0.0) | 10/52 (19.2) |
| Non-septic | 1/82 (1.2) | 18/174 (10.3) |
| Readmission within 7 days, n (%) |  |  |
| Septic | 0/6 (0.0) | 4/52 (7.7) |
| Non-septic | 5/82 (6.1) | 13/174 (7.5) |
| Length of stay (days), median [IQR] |  |  |
| Septic | 2 [1-3] | 5 [3-8] |
| Non-septic | 1 [0-4] | 5 [3-7] |
| Length of stay (days)^a^, median [IQR] |  |  |
| Septic | 2.5 [1.8-6] | 5 [3-8] |
| Non-septic | 3 [1-6] | 5 [3-7] |
| Length of antibiotic treatment (days), median [IQR] |  |  |
| Septic | 5.5 [5-6.8] | 6 [4-7.3] |
| Non-septic | 7 [5-7] | 7 [5-9.8] |

*Appendix 11:* Primary outcomes stratified according to procalcitonin. AB: Antibiotic; ICU: Intensive care unit; IQR: Interquartile range; PSI: Pneumonia severity index, PCT = procalcitonin. ^a^ Only hospitalised patients.

|  | Oral (n = 88) | Intravenous (n = 226) |
| --- | --- | --- |
| Mortality, n (%) |  |  |
| PCT ≥ 0.25 ng/mL | 3/26 (11.5) | 25/138 (18.1) |
| PCT < 0.25 ng/mL | 1/62 (1.6) | 8/88 (9.1) |
| ICU-admission, n (%) |  |  |
| PCT ≥ 0.25 ng/mL | 1/26 (3.8) | 22/138 (15.9) |
| PCT < 0.25 ng/mL | 0/62 (0.0) | 6/88 (6.8) |
| Readmission within 7 days, n (%) |  |  |
| PCT ≥ 0.25 ng/mL | 2/26 (7.7) | 12/138 (8.7) |
| PCT < 0.25 ng/mL | 3/62 (4.8) | 5/88 (5.7) |
| Length of stay (days), median [IQR] |  |  |
| PCT ≥ 0.25 ng/mL | 2 [0-4.5] | 5 [3-8] |
| PCT < 0.25 ng/mL | 1 [0-4] | 4 [2-7] |
| Length of stay (days)^a^, median [IQR] |  |  |
| PCT ≥ 0.25 ng/mL | 3.5 [1.8-6.3] | 5 [3-8] |
| PCT < 0.25 ng/mL | 3 [1-6] | 4 [2-7] |
| Length of antibiotic treatment (days), median [IQR] |  |  |
| PCT ≥ 0.25 ng/mL | 7 [5-8] | 7 [5-9] |
| PCT < 0.25 ng/mL | 7 [5-7] | 6 [5-8] |

*Appendix 12*: Microbiology results of all patients. * In the intravenous group, one blood culture tested positive for 2 micro-organisms, and one blood culture tested positive for 3 micro-organisms. ** Eleven sputum cultures tested positive for 2 micro-organisms (one in the oral group and ten in the intravenous group), and two sputum cultures tested positive for 3 micro-organisms in the intravenous group. *** Five viral swabs tested positive for 2 micro-organisms (one in the oral group and four in the intravenous group).

|  | Oral (n = 88) | Intravenous (n = 226) |
| --- | --- | --- |
| Blood cultures |  |  |
| *Number of patients* | 88 | 226 |
| *Positive, n (%)** | 5 (5.7) | 40 (17.7) |
| *Respiratory pathogen* | - | 15 |
| *Non-respiratory pathogen* | 3 | 10 |
| *Contaminant* | 2 | 18 |
| Sputum cultures |  |  |
| *Number of patients* | 34 | 95 |
| *Positive, n (%)*** | 16 (47.1) | 45 (47.4) |
| *Respiratory pathogen* | 14 | 38 |
| *Non-pathogen* | 3 | 21 |
| Viral swabs |  |  |
| *Number of patients* | 49 | 131 |
| *Positive, n (%)**** | 11 (22.4) | 35 (26.7) |
| Urine antigen tests |  |  |
| *Number of patients* | 14 | 111 |
| *Positive, n (%)* | 4 (28.6) | 21 (18.9) |
| *Legionella* | 1 | 5 |
| *Pneumococcus* | 3 | 16 |
| Atypical respiratory pathogen tests |  |  |
| *Number of patients* | 12 | 37 |
| *Positive, n (%)* | - | 5 (13.5) |
| *Chlamydophila psittaci* | - | 2 |
| *Legionella pneumophila* | - | 1 |
| *Mycoplasma pneumoniae* | - | 1 |
| *Pneumocystis jiroveci* | - | 1 |

*Appendix 13:* Microbiology results of matched patients. * One blood culture in the intravenous group tested positive for 3 micro-organisms. ** Four sputum cultures tested positive for 2 micro-organisms (one in the oral group and three in the intravenous group), and one sputum culture tested positive for 3 micro-organisms in the intravenous group. ** One viral swab in the intravenous group tested positive for 2 micro-organisms.

|  | Oral (n = 71) | Intravenous (n = 102) |
| --- | --- | --- |
| Blood cultures |  |  |
| *Number of patients* | 71 | 102 |
| *Positive, n (%)** | 3 (4.2) | 12 (11.8) |
| *Respiratory pathogen* | - | 3 |
| *Non-respiratory pathogen* | 2 | 3 |
| *Contaminant* | 1 | 8 |
| Sputum cultures |  |  |
| *Number of patients* | 29 | 44 |
| *Positive, n (%)*** | 16 (55.2) | 18 (40.9) |
| *Respiratory pathogen* | 14 | 16 |
| *Non-pathogen* | 3 | 7 |
| Viral swabs |  |  |
| *Number of patients* | 40 | 54 |
| *Positive, n (%) **** | 9 (22.5) | 12 (22.2) |
| Urine antigen tests |  |  |
| *Number of patients* | 10 | 35 |
| *Positive, n (%)* | 3 (30.0) | 4 (11.5) |
| *Legionella* | 1 | - |
| *Pneumococcus* | 2 | 4 |
| Atypical respiratory pathogen tests |  |  |
| *Number of patients* | 9 | 13 |
| *Positive, n (%)* | - | - |

*Appendix 14:* Respiratory/non-respiratory pathogens and contaminants found in the blood cultures of all patients. Respiratory pathogens are defined as micro-organisms that were interpreted by the clinician as causative for the respiratory tract infection. Non-respiratory pathogens are defined as micro-organisms that were interpreted by the clinician as causative for an infection other than respiratory. Contaminants are micro-organisms which are interpreted by the microbiologist as contamination.

|  | Oral (n = 5) | Intravenous (n = 43) |
| --- | --- | --- |
| Respiratory pathogens |  |  |
| *Escherichia coli* | - | 1 |
| *Haemophilus influenzae* | - | 3 |
| *Staphylococcus aureus* | - | 1 |
| *Streptococcus pneumoniae* | - | 10 |
| Non-respiratory pathogens |  |  |
| *Candida albicans* | - | 1 |
| *Escherichia coli* | 2 | 7 |
| *Klebsiella pneumoniae* | - | 1 |
| *Streptococcus dysgalactiae* | 1 | 1 |
| Contaminants |  |  |
| *Lactobacillus species* | - | 1 |
| *Paenibacillus species* | 1 | - |
| *Staphylococcus capitis* | - | 2 |
| *Staphylococcus epidermidis* | 1 | 5 |
| *Staphylococcus hominis* | - | 9 |
| *Streptococcus mitis* | - | 1 |

*Appendix 15:* Respiratory pathogens, non-respiratory pathogens and contaminants found in blood cultures of matched patients. Respiratory pathogens are defined as micro-organisms that were interpreted by the clinician as causative for the respiratory tract infection. Non-respiratory pathogens are defined as micro-organisms that were interpreted by the clinician as causative for an infection other than respiratory. Contaminants are micro-organisms which are interpreted by the microbiologist as contamination.

|  | Oral (n = 3) | Intravenous (n = 14) |
| --- | --- | --- |
| Respiratory pathogens |  |  |
| *Haemophilus influenzae* | - | 1 |
| *Streptococcus pneumoniae* | - | 2 |
| Non-respiratory pathogens |  |  |
| *Candida albicans* | - | 1 |
| *Escherichia coli* | 2 | 2 |
| Contaminants |  |  |
| *Lactobacillus species* | - | 1 |
| *Paenibacillus species* | 1 | - |
| *Staphylococcus capitis* | - | 2 |
| *Staphylococcus epidermidis* | - | 1 |
| *Staphylococcus hominis* | - | 4 |

*Appendix 16:* Respiratory pathogens and non-pathogens found in sputum cultures in all patients. Respiratory pathogens are defined as micro-organisms that were interpreted by the clinician as causative for the respiratory tract infection. Non pathogens are defined as micro-organisms that were found but were not seen as causative for the respiratory tract infection.

|  | Oral (n = 17) | Intravenous (n = 59) |
| --- | --- | --- |
| Respiratory pathogens |  |  |
| *Acinetobacter species* | - | 1 |
| *Aspergillus fumigatus* | - | 1 |
| *Escherichia coli* | - | 3 |
| *Haemophilus influenzae* | 8 | 9 |
| *Haemophilus parainfluenzae* | - | 2 |
| *Klebsiella pneumoniae* | - | 2 |
| *Moraxella catarrhalis* | 2 | 3 |
| *Proteus mirabilis* | - | 1 |
| *Proteus vulgaris* | 1 | - |
| *Pseudomonas aeruginosa* | 2 | 3 |
| *Serratia marcescens* | - | 1 |
| *Serratia odorifera* | - | 1 |
| *Staphylococcus aureus* | - | 3 |
| *Streptococcus pneumoniae* | 1 | 6 |
| *Streptococcus pyogenes* | - | 2 |
| Non-pathogens |  |  |
| *Aspergillus fumigatus* | - | 3 |
| *Candida albicans* | - | 5 |
| *Candida dubliniensis* | - | 1 |
| *Candida tropicalis* | - | 2 |
| *Escherichia coli* | - | 2 |
| *Enterobacter cloacae complex* | 1 | - |
| *Mycobacterium paragordonae* | - | 1 |
| *Pseudomonas aeruginosa* | 1 | - |
| *Staphylococcus aureus* | 1 | 6 |
| *Stenotrophomonas maltophilia* | - | 1 |

*Appendix 17:* Respiratory pathogens and non-pathogens found in sputum cultures in matched patients. Respiratory pathogens are defined as micro-organisms that were interpreted by the clinician as causative for the respiratory tract infection. Non pathogens are defined as micro-organisms that were found but were not seen as causative for the respiratory tract infection.

|  | Oral (n = 17) | Intravenous (n = 23) |
| --- | --- | --- |
| Respiratory pathogens |  |  |
| *Acinetobacter species* | - | 1 |
| *Aspergillus fumigatus* | - | 1 |
| *Haemophilus influenzae* | 8 | 4 |
| *Haemophilus parainfluenzae* | - | 1 |
| *Klebsiella pneumoniae* | - | 1 |
| *Moraxella catarrhalis* | 2 | 2 |
| *Proteus vulgaris* | 1 | - |
| *Pseudomonas aeruginosa* | 2 | 3 |
| *Streptococcus pneumoniae* | 1 | 2 |
| *Streptococcus pyogenes* | - | 1 |
| Non-pathogens |  |  |
| *Aspergillus fumigatus* | - | 1 |
| *Candida albicans* | - | 1 |
| *Escherichia coli* | - | 1 |
| *Enterobacter cloacae complex* | 1 | - |
| *Mycobacterium paragordonae* | - | 1 |
| *Pseudomonas aeruginosa* | 1 | - |
| *Staphylococcus aureus* | 1 | 2 |
| *Stenotrophomonas maltophilia* | - | 1 |

*Appendix 18:* Respiratory pathogens found in viral swabs in all patients.

|  | Oral (n = 12) | Intravenous (n = 39) |
| --- | --- | --- |
| Respiratory pathogens |  |  |
| *Adenovirus* | - | 1 |
| *Coronavirus HKU1* | 1 | 2 |
| *Coronavirus NL63* | - | 1 |
| *Human metapneumovirus* | 1 | 3 |
| *Influenza A* | 3 | 11 |
| *Influenza B* | - | 1 |
| *Parainfluenzavirus 1* | 2 | 2 |
| *Parainfluenzavirus 3* | - | 3 |
| *Parainfluenzavirus 4* | - | 1 |
| *Rhinovirus* | 3 | 10 |
| *Respiratory syncytial virus* | 2 | 4 |

*Appendix 19:* Respiratory pathogens found in viral swabs in matched patients.

|  | Oral (n = 9) | Intravenous (n = 13) |
| --- | --- | --- |
| Respiratory pathogens |  |  |
| *Adenovirus* | - | 1 |
| *Coronavirus NL63* | - | 1 |
| *Human metapneumovirus* | 1 | 2 |
| *Influenza A* | 3 | 2 |
| *Parainfluenzavirus 1* | 2 | - |
| *Parainfluenzavirus 3* | - | 1 |
| *Rhinovirus* | 2 | 4 |
| *Respiratory syncytial virus* | 1 | 2 |

*Appendix 20:* Antibiotic regimen of all patients at start of treatment. AB: Antibiotic; ED: Emergency department; IV: Intravenous. * Amoxicillin (n = 2), Ceftriaxone (n = 7) and Ceftriaxone + Tobramycine (n = 1).

|  | Oral (n = 88) | Intravenous (n = 226) |
| --- | --- | --- |
| AB regimen |  |  |
| *Amoxicillin* | 38 | 74 |
| *Amoxicillin + Ceftriaxone + Metronidazole* | - | 1 |
| *Amoxicillin + Ciprofloxacin* | - | 3 |
| *Amoxicillin clavulanic acid* | 9 | 15 |
| *Benzyl penicillin + Ceftazidime* | - | 2 |
| *Ceftazidime* | - | 2 |
| *Ceftazidime + Ciprofloxacin* | - | 1 |
| *Ceftazidime + Flucloxacillin* | - | 1 |
| *Ceftriaxone* | - | 59 |
| *Ceftriaxone + Ciprofloxacin* | - | 25 |
| *Ceftriaxone + Doxycycline* | - | 1 |
| *Ceftriaxone + Metronidazole* | - | 2 |
| *Ceftriaxone + Tobramycin* | - | 2 |
| *Ceftriaxone + Vancomycin* | - | 1 |
| *Cefuroxime* | - | 30 |
| *Cefuroxime + Ciprofloxacin* | - | 1 |
| *Cefuroxime + Tobramycin* | - | 2 |
| *Ciprofloxacin* | 1 | 2 |
| *Clindamycin* | - | 1 |
| *Doxycycline* | 22 | - |
| *Imipenem/cilastatin* | - | 1 |
| *Flucloxacillin* | 1 | - |
| *Moxifloxacin* | 16 | - |
| *Trimethoprim/sulfamethoxazole* | 1 | - |
| Single IV administration at ED | 10* | - |

*Appendix 21:* Antibiotic regimen of matched patients at start of treatment. AB: Antibiotic; ED: Emergency department; IV: Intravenous. * Amoxicillin (n = 2), Ceftriaxone (n = 6) and Ceftriaxone + Tobramycine (n = 1).

|  | Oral (n = 71) | Intravenous (n = 102) |
| --- | --- | --- |
| AB regimen |  |  |
| *Amoxicillin* | 31 | 38 |
| *Amoxicillin + Ciprofloxacin* | - | - |
| *Amoxicillin clavulanic acid* | 6 | 11 |
| *Benzyl penicillin + Ceftazidime* | - | 1 |
| *Ceftazidime* | - | 2 |
| *Ceftazidime + Flucloxacillin* | - | 1 |
| *Ceftriaxone* | - | 27 |
| *Ceftriaxone + Ciprofloxacin* | - | 3 |
| *Ceftriaxone + Tobramycin* | - | 2 |
| *Cefuroxime* | - | 15 |
| *Ciprofloxacin* | 1 | - |
| *Clindamycin* | - | 1 |
| *Doxycycline* | 19 | - |
| *Imipenem/cilastatin* | - | 1 |
| *Flucloxacillin* | 1 | - |
| *Moxifloxacin* | 13 | - |
| Single IV administration at ED | 9* | - |

*Appendix 22:* Switches in antibiotic (AB) regimen in all patients. AB: Antibiotic; IQR: Interquartile range; IV: Intravenous. * In this calculation, nineteen additional patients were added because these patients switched to oral AB in a later switch than the first switch.

|  | Oral (n = 88) | Intravenous (n = 226) |
| --- | --- | --- |
| Switch in AB treatment, n (%) | 16 (18.2) | 180 (79.6) |
| *First switch was to oral AB* |  |  |
| *Switch to oral treatment with same AB* | - | 74 |
| *Switch to oral treatment with different AB* | 9 | 74 |
| *First switch was to IV AB* |  |  |
| *Switch to IV treatment with same AB* | 2 | - |
| *Switch to IV treatment with different AB* | 5 | 32 |
| *Therapeutic switch, n (%)* | 15 (93.8) | 25 (13.9) |
| Time until first AB switch (days), median [IQR] | 2 [1-3] | 2 [1-3] |
| *Time until switch to oral AB, median [IQR]** | - | 2 [1-3] |
| *Time until switch to IV AB, median [IQR]* | 1 [1-1] | - |

*Appendix 23:* Switches in antibiotic (AB) regimen in matched patients. AB: Antibiotic; IQR: Interquartile range; IV: Intravenous. * In this calculation, nine additional patients were added because these patients switched to oral AB in a later switch than the first switch.

|  | Oral (n = 71) | Intravenous (n = 102) |
| --- | --- | --- |
| Switch in AB treatment, n (%) | 14 (19.7) | 87 (85.3) |
| *First switch was to oral AB* |  |  |
| *Switch to oral treatment with same AB* | - | 36 |
| *Switch to oral treatment with different AB* | 9 | 39 |
| *First switch was to IV AB* |  |  |
| *Switch to IV treatment with same AB* | 2 | - |
| *Switch to IV treatment with different AB* | 3 | 12 |
| *Therapeutic switch, n (%)* | 13 (92.9) | 13 (12.7) |
| Time until first AB switch (days), median [IQR] | 2 [1-3] | 2 [1-3] |
| *Time until switch to oral AB, median [IQR]** | - | 2 [1-3] |
| *Time until switch to IV AB, median [IQR]* | 1 [1-1] | - |

*Appendix 24:* Antibiotic regimen per Pneumonia severity index (PSI) class in all patients.

|  | Oral (n = 88) | Intravenous (n = 226) |
| --- | --- | --- |
| PSI III, n (%) |  |  |
| *Amoxicillin* | 15/33 (45.5) | 19/43 (44.2) |
| *Amoxicillin/clavulanic acid* | 2/33 (6.1) | - |
| *Benzyl penicillin + Ceftazidime* | - | 2/43 (4.7) |
| *Ceftazidime* | - | 2/43 (4.7) |
| *Ceftriaxone* | - | 6/43 (14.0) |
| *Ceftriaxone + Ciprofloxacin* | - | 5/43 (11.6) |
| *Ceftriaxone + Doxycycline* | - | 1/43 (2.3) |
| *Cefuroxime* | - | 6/43 (14.0) |
| *Ciprofloxacin* | - | 1/43 (2.3) |
| *Doxycycline* | 9/33 (27.3) | - |
| *Flucloxacillin* | 1/33 (3.0) | - |
| *Imipenem/cilastatine* | - | 1/43 (2.3) |
| *Moxifloxacin* | 5/33 (15.2) | - |
| *Trimethoprim/sulfamethoxazole* | 1/33 (3.0) | - |
| PSI IV, n (%) |  |  |
| *Amoxicillin* | 22/50 (44.0) | 46/121 (38.0) |
| *Amoxicillin + Ciprofloxacin* | - | 3/121 (2.5) |
| *Amoxicillin/clavulanic acid* | 5/50 (10.0) | 12/121 (9.9) |
| *Ceftazidime + Ciprofloxacin* | - | 1/121 (0.8) |
| *Ceftazidime + Flucloxacillin* | - | 1/121 (0.8) |
| *Ceftriaxone* | - | 28/121 (23.1) |
| *Ceftriaxone + Ciprofloxacin* | - | 8/121 (6.6) |
| *Ceftriaxone + Tobramycin* | - | 1/121 (0.8) |
| *Cefuroxime* | - | 17/121 (14.0) |
| *Cefuroxime + Ciprofloxacin* | - | 1/121 (0.8) |
| *Cefuroxime + Tobramycin* | - | 2/121 (1.7) |
| *Ciprofloxacin* | 1/50 (2.0) | - |
| *Clindamycin* | - | 1/121 (0.8) |
| *Doxycycline* | 12/50 (24.0) | - |
| *Moxifloxacin* | 10/50 (20.0) | - |
| PSI V, n (%) |  |  |
| *Amoxicillin* | 1/5 (20.0) | 9/62 (14.5) |
| *Amoxicillin + Ceftriaxone + Metronidazole* | - | 1/62 (1.6) |
| *Amoxicillin/clavulanic acid* | 2/5 (40.0) | 3/62 (4.8) |
| *Ceftriaxone* | - | 25/62 (40.3) |
| *Ceftriaxone + Ciprofloxacin* | - | 12/62 (19.4) |
| *Ceftriaxone + Metronidazole* | - | 2/62 (3.2) |
| *Ceftriaxone + Tobramycin* | - | 1/62 (1.6) |
| *Ceftriaxone + Vancomycin* | - | 1/62 (1.6) |
| *Cefuroxime* | - | 7/62 (11.3) |
| *Ciprofloxacin* | - | 1/62 (1.6) |
| *Doxycycline* | 1/5 (20.0) | - |
| *Moxifloxacin* | 1/5 (20.0) | - |

*Appendix 25:* Antibiotic regimen per Pneumonia severity index (PSI) class in matched patients.

|  | Oral (n = 71) | Intravenous (n = 102) |
| --- | --- | --- |
| PSI III, n (%) |  |  |
| *Amoxicillin* | 10/22 (45.5) | 12/26 (46.2) |
| *Amoxicillin/clavulanic acid* | 1/22 (4.5) | - |
| *Benzyl penicillin + Ceftazidime* | - | 1/26 (3.8) |
| *Ceftazidime* | - | 2/26 (7.7) |
| *Ceftriaxone* | - | 4/26 (15.4) |
| *Ceftriaxone + Ciprofloxacin* | - | 1/26 (3.8) |
| *Cefuroxime* | - | 5/26 (19.2) |
| *Doxycycline* | 7/22 (31.8) | - |
| *Flucloxacillin* | 1/22 (4.5) | - |
| *Imipenem/cilastatine* | - | 1/26 (3.8) |
| *Moxifloxacin* | 3/22 (13.6) | - |
| PSI IV, n (%) |  |  |
| *Amoxicillin* | 20/44 (45.5) | 23/62 (37.1) |
| *Amoxicillin/clavulanic acid* | 3/44 (6.8) | 9/62 (14.5) |
| *Ceftazidime + Flucloxacillin* | - | 1/62 (1.6) |
| *Ceftriaxone* | - | 19/62 (30.6) |
| *Ceftriaxone + Tobramycin* | - | 1/62 (1.6) |
| *Cefuroxime* | - | 8/62 (12.9) |
| *Ciprofloxacin* | 1/44 (2.3) | - |
| *Clindamycin* | - | 1/62 (1.6) |
| *Doxycycline* | 11/44 (25.0) | - |
| *Moxifloxacin* | 9/44 (20.5) | - |
| PSI V, n (%) |  |  |
| *Amoxicillin* | 1/5 (20.0) | 3/14 (21.4) |
| *Amoxicillin/clavulanic acid* | 2/5 (40.0) | 2/14 (14.3) |
| *Ceftriaxone* | - | 4/14 (28.6) |
| *Ceftriaxone + Ciprofloxacin* | - | 2/14 (14.3) |
| *Ceftriaxone + Tobramycin* | - | 1/14 (7.1) |
| *Cefuroxime* | - | 2/14 (14.3) |
| *Doxycycline* | 1/5 (20.0) | - |
| *Moxifloxacin* | 1/5 (20.0) | - |
